# Reflex Anuria Precipitated by Unknown Cause: A Case Report

**DOI:** 10.7759/cureus.19815

**Published:** 2021-11-22

**Authors:** Thrshegan Theivendrampillai, Marta Chacon Garcia, Sarosh Janardanan

**Affiliations:** 1 Urology, Royal Surrey County Hospital, Guildford, GBR

**Keywords:** hydronephrosis, bilateral nephrostomy tube, urology, reflex anuria, acute kidney injury

## Abstract

Reflex anuria is a rare phenomenon that is caused by trauma or irritation to one kidney or ureter. It has been described in unilateral ureteric obstruction and after surgical procedures involving the ureters and bladder. Here we present a case of a 33-year-old female, presenting with right-sided loin to groin pain, which eventually lead to oliguria and required bilateral nephrostomies as retrograde stent insertions failed. Although an etiology was not identified, we postulate that unilateral ureteric obstruction triggered reflex anuria. Although reflex anuria is a rare presentation, it should be included in the differential diagnosis once all other causes of AKI have been ruled out. Furthermore, this report will show that bilateral nephrostomies should be considered as a viable treatment option for reflex anuria.

## Introduction

Causes of acute kidney injury (AKI) are classified into three categories: prerenal, intrarenal and postrenal components. Typically, in unilateral ureteric obstruction with the presence of a normal functioning contralateral kidney, renal function is expected to be normal or only mildly impaired. However, Hull et al. described another rare entity called reflex anuria as a cause of AKI in patients with unilateral ureteric obstruction [1].

Reflex anuria is defined as “cessation of urine output from both kidneys in response to irritation or trauma to one kidney or its ureter or severely painful stimuli to other pelvic organs” [1]. This rare phenomenon, which is not fully understood is thought to cause either reflex vasoconstriction of arterioles leading to reduced renal function or reflex spasm of both ureters causing a functional obstruction. It has been commonly associated with unilateral ureteric obstruction [2] and after surgical procedures involving the ureters and the bladder [3]. Furthermore, reflex anuria causing AKI has also been explained in instances where ureteric manipulation was performed during pelvic surgery in the absence of any ureteral obstruction [4].

We present a case of reflex anuria precipitated by an unknown cause. This report will add to what little is known about this phenomenon and provide greater insight into the presentation, diagnosis and treatment of reflex anuria.

## Case presentation

A 33-year-old female with a past medical history including meningitis, epilepsy and migraines, presented with a one-day history of right-sided sharp loin to groin pain, with associated nausea and vomiting. Physical examination revealed right flank tenderness and the patient’s vital signs were stable.

Laboratory evaluation was unremarkable with a normal serum creatinine of 78 micromol/L (0.88mg/dL) and an estimated glomerular filtration rate (eGFR) of 87 mL/min. Urine dipstick was negative for leukocytes, proteins and nitrites, but positive for blood and urine output was more than 60 mL/hr. All these features were consistent with a right-sided renal stone and therefore the patient was subsequently given one dose of Diclofenac per rectum and oral morphine for pain relief.

**Figure 1:**
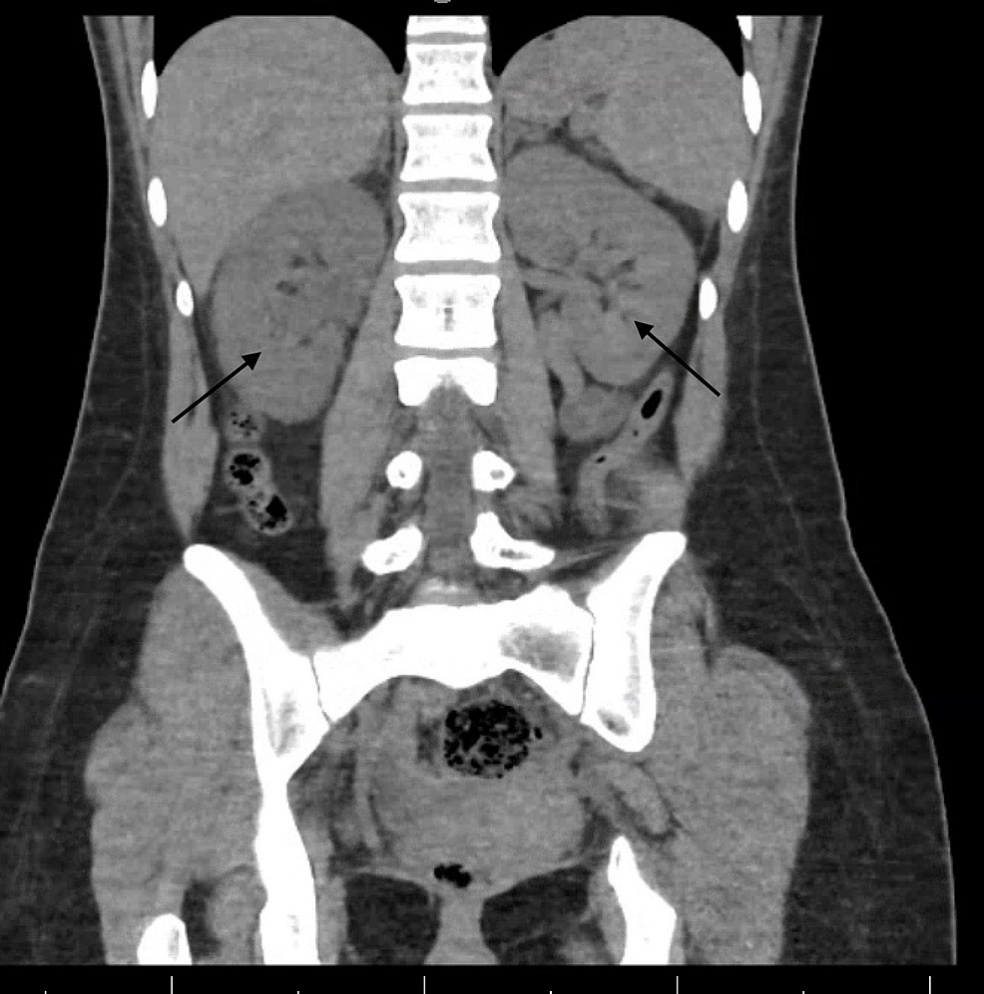
Coronal view of CT abdomen pelvis without contrast showing mild right hydronephrosis. Note the pelvicalyceal dilatation of the right kidney, whereas the left kidney is completely decompressed

Non-contrast CT abdomen pelvis (Figure 1) revealed mild right-sided hydronephrosis and hydroureter restricted to the proximal ureters. Although no obvious renal tract calculus or obstructing lesion was evident, there was some residual fullness secondary to edema suggesting that a small right-sided calculus may have passed.

On the second day of admission, the patient went on to develop bilateral flank pain, more pronounced on the left this time. There was a significant rise in serum creatinine level from 78 to 266 micromol/L (3.01 mg/dL) with poor urine output, only passing 235 mL in the first 15 hours of the day. As a result, the patient was catheterized and noted to be oliguric with 0ml urine output for six hours despite aggressive fluid resuscitation. During day 2 of admission, the patient had a positive 2,086 mL fluid balance. After extensive clinical evaluation by the Medical team with also the Nephrology team being consulted, no prerenal or intrinsic renal cause of AKI was identified. In our patient, pre-renal causes were immediately ruled out in the absence of dehydration, blood loss, heart failure and sepsis. An autoimmune screen was carried out which was negative for ANA, ANCA and C3, making autoimmune disease less likely.

**Figure 2:**
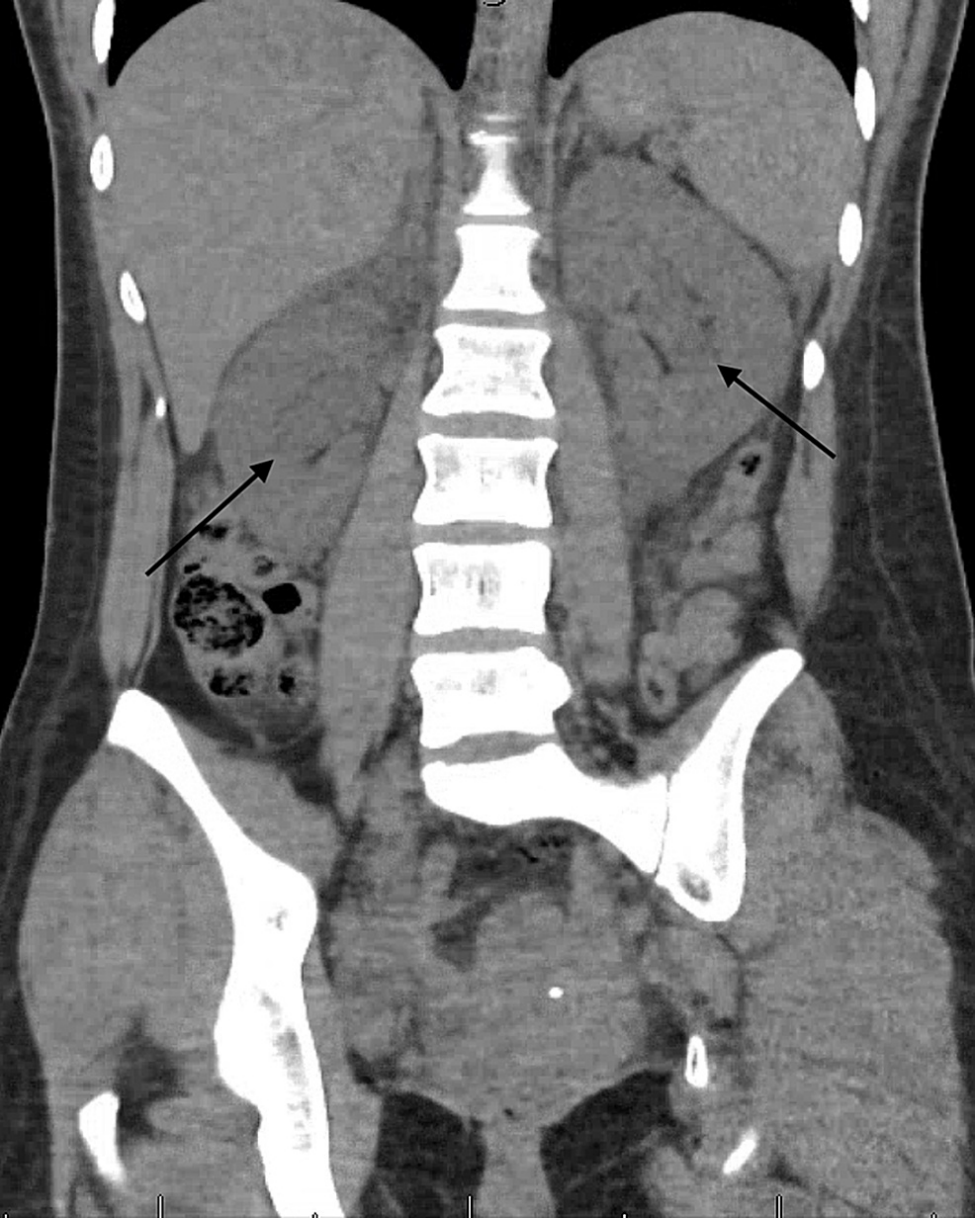
Coronal view of non-contrast CT abdomen pelvis showing bilateral hydronephrosis

As soon as the deterioration in renal function was noted, repeat non-contrast CT abdomen pelvis (Figure 2) was performed, which now showed mild bilateral hydronephrosis suggesting obstructive uropathy. However, once again, no mechanical obstruction or stricture on either side was identified.

**Figure 3:**
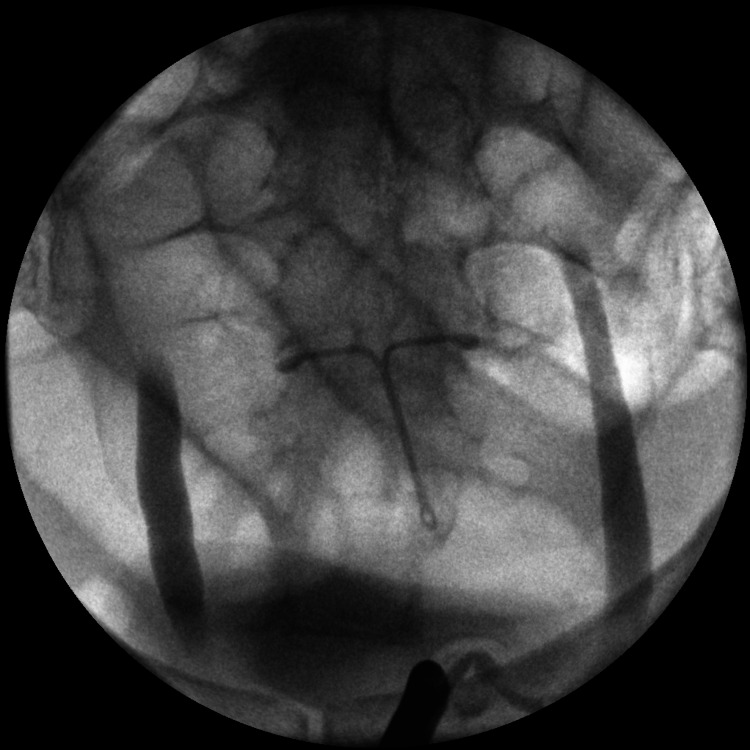
Retrograde study showing no strictures or mechanical obstruction. Note the intrauterine device and dilated ureters on the figure.

**Figure 4:**
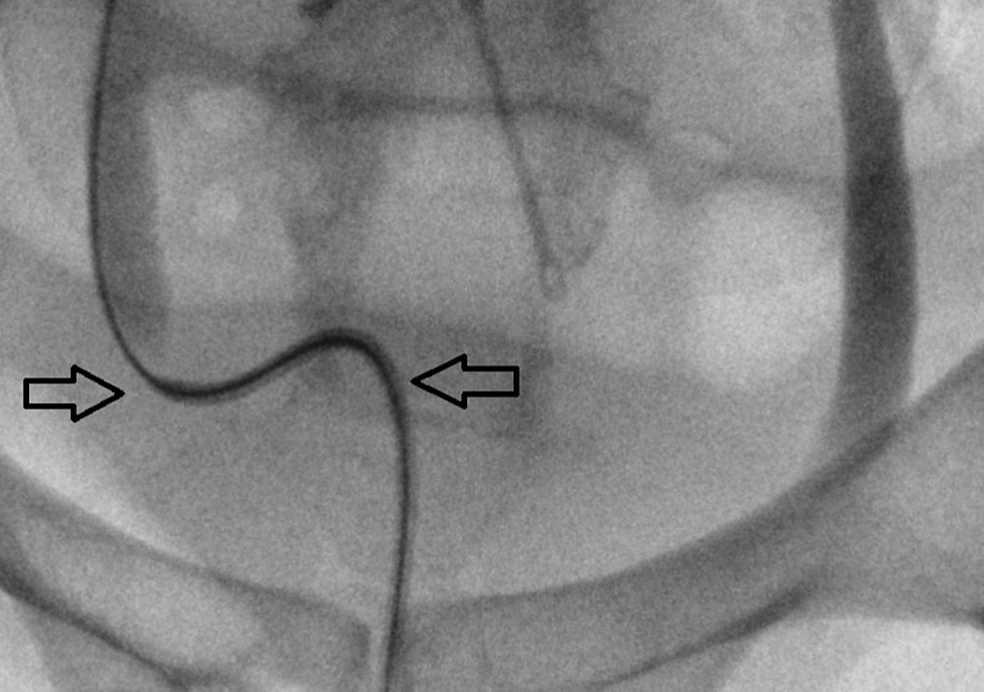
Retrograde access beyond the ureteric orifice was not possible even with a 6 French ureteric catheter (left arrow) over a guidewire. Proximally to this point (right arrow), the ureter was noted to be dilated.

**Figure 5:**
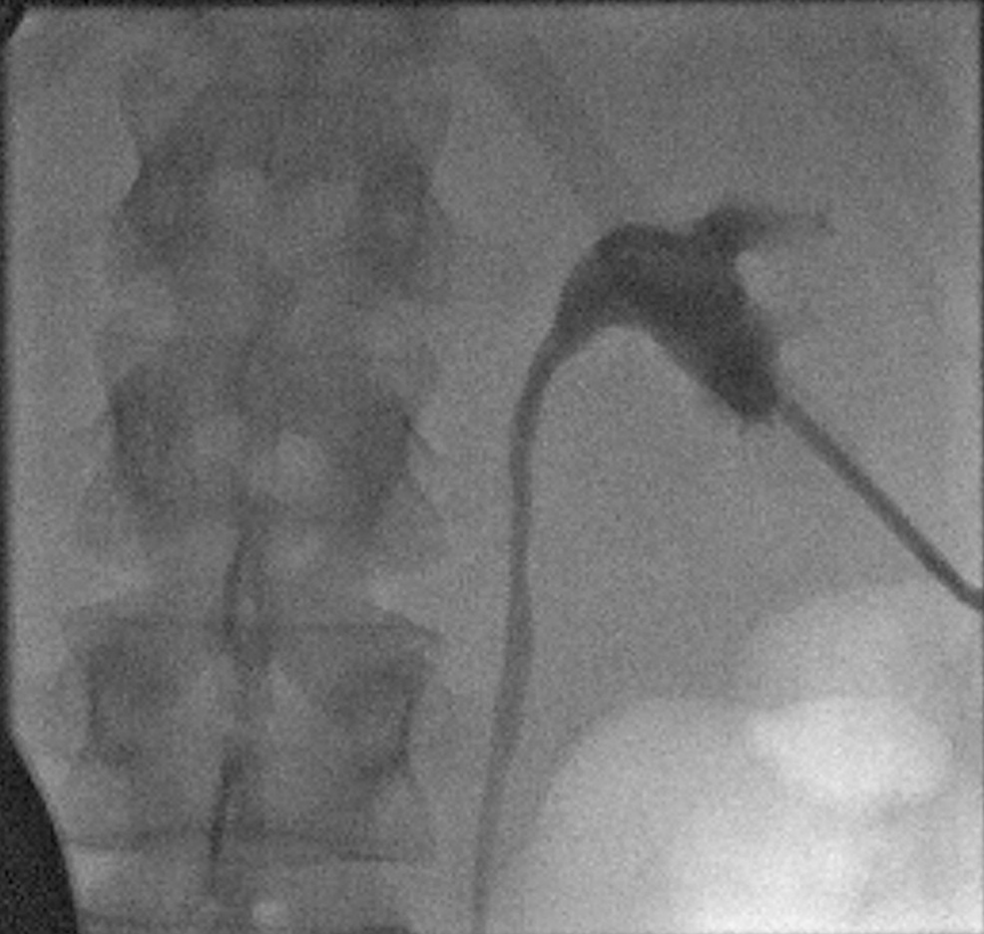
Nephrostogram showing no filling defects

Rigid cystoscopy with retrograde pyelogram (Figure 3) was performed as an emergency in a surgical theatre, which revealed no evidence of stricture or mechanical obstruction in the distal ureters. We were unable to pass a 6 French ureteric catheter beyond both ureteric orifices for reasons that can not be explained (Figure 4), despite showing dilated ureters. This made having retrograde pictures of the whole system impossible with contrast draining into the bladder. This further prevented instrumentation on both sides including a failure to stent the system, despite a normal bladder being noted on cystoscopy. As a result, bilateral nephrostomies were inserted under ultrasound guidance by Interventional Radiologists and the position was confirmed using contrast and x-ray. A nephrostogram (Figure 5) was performed, which showed no filling defects of the ureters suggesting that there was no mechanical obstruction.

 

 

**Figure 6:**
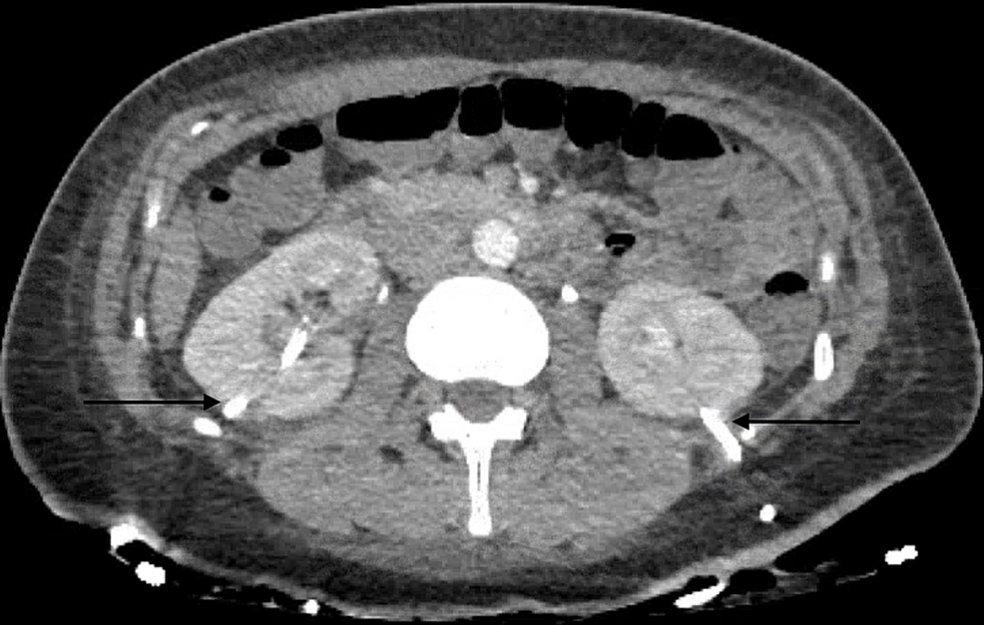
Axial view of CT urogram showing no hydronpehrosis while having the nephrostomoies clamped (left and right arrow)

**Figure 7:**
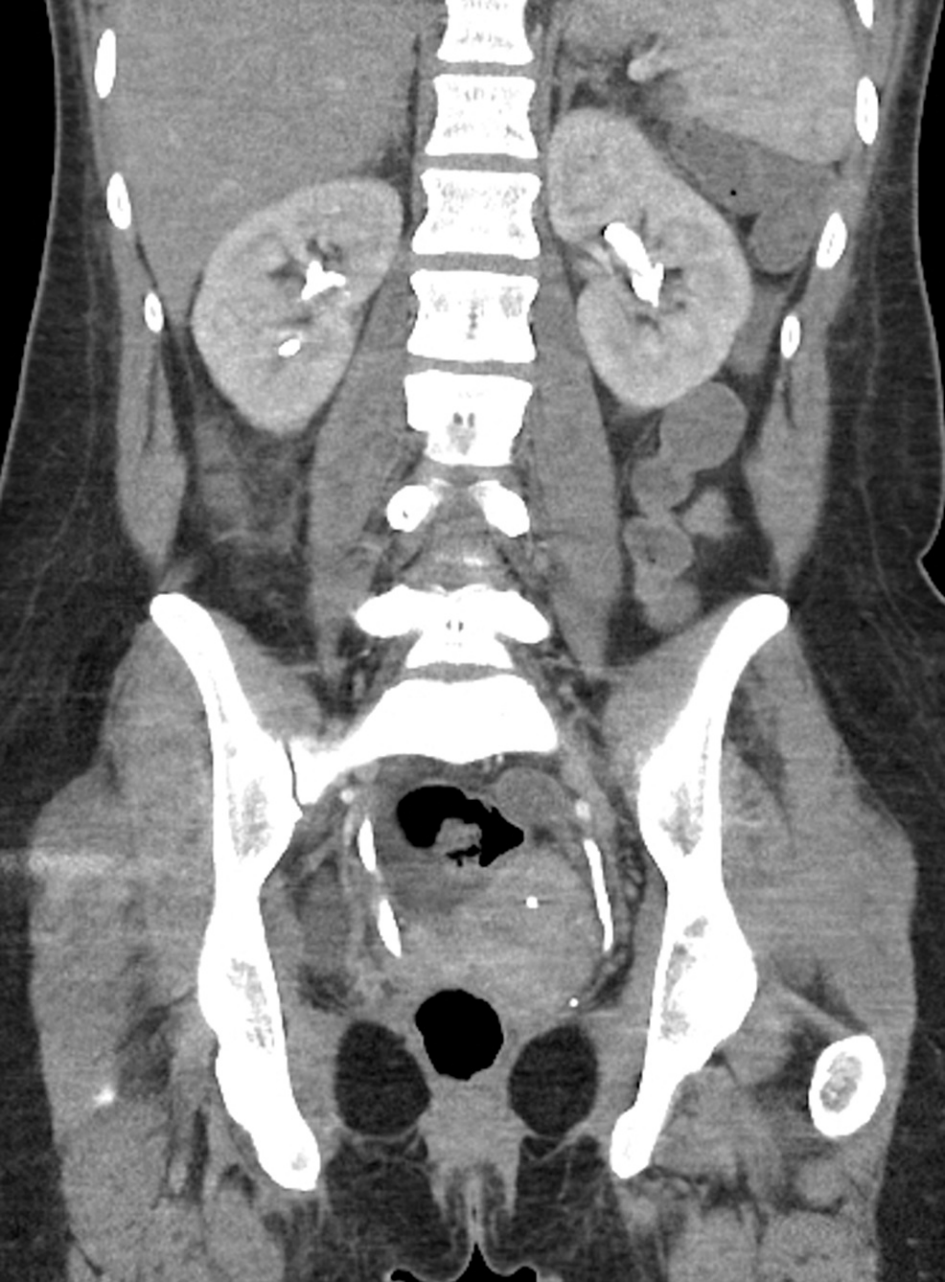
Coronal view of CT urogram showing no evidence of hydronephrosis on either side with normal contrast excretion

The next 24 hours (day 3) showed an improving urine output of 3.6L with a reduction in the serum creatinine level to 166 micromol/L (1.88 mg/dL). The patient continued to improve over the next three days with a steady decline of serum creatinine level to back to baseline (72 micromol/L [0.81 mg/dL]) with good urine output, returning back to a euvolemic status. A subsequent CT urogram, while having the nephrostomies clamped (Figure 6). Figure 7 showed unremarkable appearances of both kidneys with no evidence of hydronephrosis.

 

The nephrostomy tubes were clamped and as the patient remained asymptomatic with good urine output and normal renal function, these were removed. The patient was subsequently discharged on day 9 of admission.

**Figure 8:**
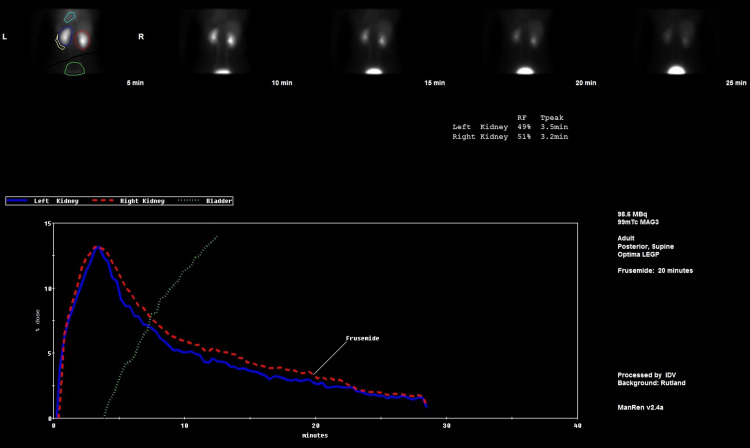
MAG3 renogram showing bilateral normal function and drainage

The patient was followed up six weeks later with a MAG3 (Mercapto-acetyl triglycine) renogram (Figure 8), which was normal and revealed no evidence of obstruction. The patient remained well and asymptomatic following 12 months after discharge. Of note, the patient had a 24-hour urine electrolyte screen as an outpatient, which was normal.

## Discussion

We report a case of reflex anuria of unknown cause. In our patient, pre-renal causes were immediately ruled out in the absence of dehydration, blood loss, heart failure and sepsis. There were no signs of hypotension or sepsis effectively ruling out acute tubular necrosis. NSAIDs were stopped immediately on day 2 of admission as per Nephrology team and acute interstitial nephritis (AIN) was ruled out in the presence of bilateral hydronephrosis. An autoimmune screen was carried out which was negative making autoimmune renal disease less likely. Although bilateral hydronephrosis was evident, an obvious obstruction could not be identified.

Although the main differential diagnosis upon admission was a right-sided ureteric stone, CT scans could not identify any obvious ureteral lesion or calculus. It is very unusual for a right-sided ureteric stone, which had likely passed, to cause the significant AKI that we saw in this patient. Patients presenting with unilateral ureteral obstruction commonly have a normal or mildly impaired renal function, in the presence of a normally functioning contralateral kidney. Furthermore, one would also expect that the mild right hydronephrosis would have resolved after the passage of the stone. However, in our case, the patient’s renal function deteriorated suddenly with new-onset bilateral flank pain accompanied by bilateral hydronephrosis in the absence of mechanical obstruction.

A review of medical literature revealed a few cases of AKI in the presence of a normally functioning contralateral kidney - this was called reflex anuria. Reflex anuria was defined by Hull et al. as “cessation of urine output from both kidneys in response to irritation or trauma to one kidney or its ureter or severely painful stimuli to other pelvic organs” in 1980 [1].

The pathogenesis of the reflex anuria is not fully understood but two hypotheses have gained acceptance. The first mechanism is called the “Neurovascular reflex,” which suggests that unilateral renal parenchymal or ureteric damage triggers reflex vasoconstriction of both kidneys’ intrarenal arterioles leading to a decrease in glomerular filtration rate resulting in AKI. Kervancioglu et al. described a case of reflex anuria after transarterial-embolization of a renal tumor that supports this mechanism [5]. Anuria developed immediately after embolization and resolved 74 hours following the procedure. They postulated that reflux anuria was stimulated by the occluded blood vessels causing ischemia of the renal tissue of the embolized kidney.

The second mechanism suggests that pain caused by stimulus/irritation of one kidney or ureter triggers a reflex spasm of both ureters causing functional obstruction with hydronephrosis. 

The diagnosis of reflex anuria is based on three criteria [6]:

· A normal contralateral kidney, which retains normal function soon after the disease-causing non‐functional kidney has been treated.

· Subsequent investigation of the normal contralateral kidney shows that a pathological process is unlikely to have caused its loss of function.

· Surgical intervention to the contralateral “shutdown” kidney does not result in the return of function in either of the kidneys.

Reflex anuria has been seen in patients with unilateral ureteric calculus and after surgical procedures involving the ureters and bladder. Literature also describes reflex anuria seen after stenting in colorectal and gynecological surgeries and has been reported in a patient after the acute cardiac event [7].

After extensive reviewing of medical literature, only a few cases are reported in females. Khan et al. study revealed that all 20 patients who developed reflex anuria secondary to unilateral ureteral calculus were men [2]. They hypothesized that the dilatory effect of progesterone may help to facilitate the passage of ureteral stones in females [8].

The initial treatment of reflex anuria consists of correction of fluid and electrolyte imbalance and monitoring fluid balance. Whilst most case reports showed that reflex anuria was treated successfully with bilateral stenting of the ureters, Kanno et al. reported a case of reflex anuria secondary to retrograde pyelography, which was treated promptly with hemodialysis [9]. Nephrostomies are infrequently used for the treatment of reflex anuria and have only been used to treat one of the 20 patients described in the case series by Khan et al. [2].

As seen in Figures 3, 4, the guidewire is seen in the ureter but a six French catheter was not able to pass through the ureteric orifice, despite the bladder appearing normal on cystoscopy and all extensive imaging studies showing no obstruction. As a result, the whole ureteral span could not be visualized. There is indeed proximal dilatation of the ureters immediately beyond the ureteric orifice, which would be in keeping with the obstructive uropathy clinical picture that we see throughout the case.

As a result, the patient had bilateral nephrostomies inserted, which resolved the AKI promptly. Whilst the patient had no urine output for a total of six hours, the patient went into the diuretic phase shortly after having the bilateral nephrostomies with a urine output of 3.6 L over the first 24 hours, in association with declining serum creatinine. The nephrostogram done at the time of the nephrostomy, along with the CT urogram done four days later, confirmed no mechanical obstruction, yet the patient had bilateral hydronephrosis. We further confirmed this by performing a MAG3 renogram six weeks later, which did not show any obstruction of both kidneys.

Considering these presented facts, if a permanent physical obstruction was present, upon clamping and removal of the nephrostomies, the patient’s renal function would have deteriorated, and the patient would have continued to be symptomatic or would have returned back to the hospital again with a significant AKI. However, the patient had remained well and asymptomatic following 12 months with normal renal function. Second, if a physical obstruction were present, the CT urogram, the nephrostogram or retrograde pyelogram would have identified a filling defect of the ureters.

In this patient, we postulate that although a right urinary calculus was not seen on the CT scans, considering the initial presentation of right-sided loin to groin pain with microscopic haematuria; this episode of reflex anuria could be attributed to a right urinary calculus. Ultimately, in our case report, it is difficult to know the exact pathophysiological mechanism responsible for this reflex anuria, yet to the best of our knowledge, there is no definitive mechanism to explain this rare phenomenon [1-9].

## Conclusions

Reflex anuria can be precipitated by many causes including unilateral ureteric calculus. Although reflex anuria is a rare presentation especially in female patients, it should be included in the differential diagnosis once all other causes of AKI have been ruled out.

We also recommend that clinicians should consider bilateral nephrostomies as a viable treatment option for patients with reflex anuria alongside bilateral stenting and hemodialysis, especially in the presence of hydronephrosis.
